# Supplementary figures and images for: Carotenoid assembly regulates quinone diffusion and the Roseiflexus castenholzii reaction center-light harvesting complex architecture
Source: eLife. 2023 Sep 22;12:e88951. doi: 10.7554/eLife.88951 (PMC10516601; doi:10.7554/eLife.88951)

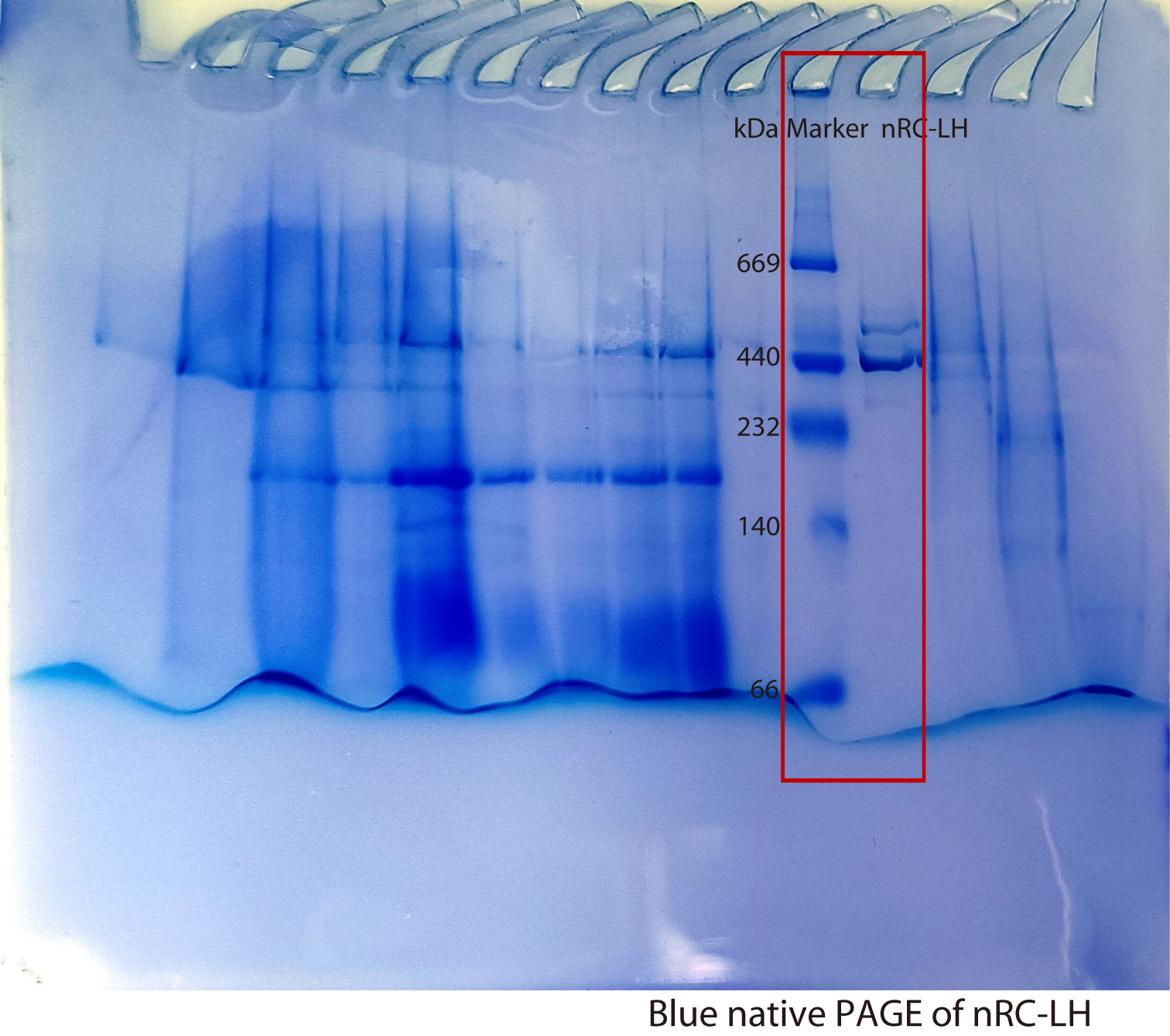

Supplement: Figure 1—figure supplement 1—source data 1. [file elife-88951-fig1-figsupp1-data1.zip › Figure 1-figure supplement 1-source data 1 with label.tif]

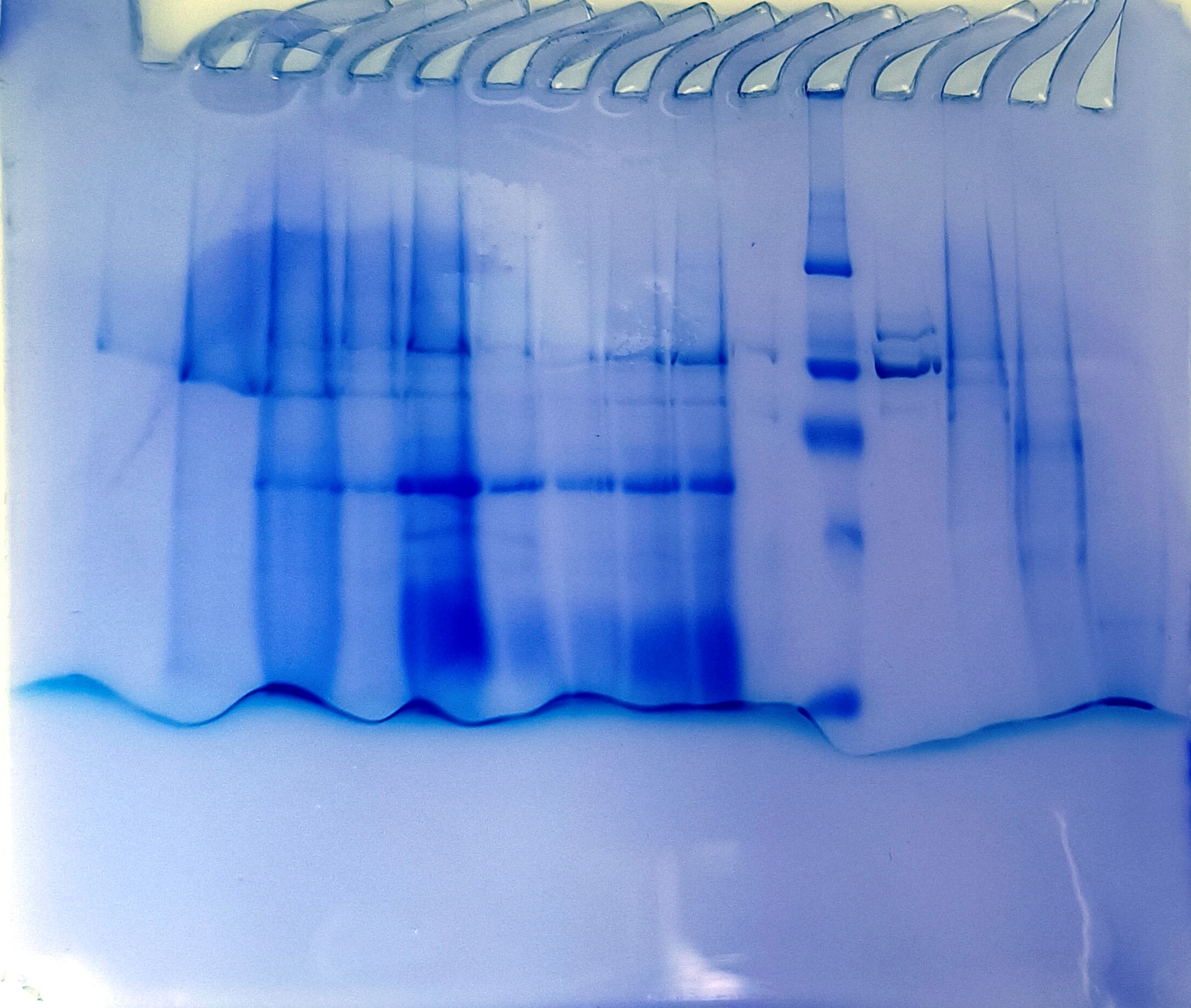

Supplement: Figure 1—figure supplement 1—source data 1. [file elife-88951-fig1-figsupp1-data1.zip › Figure 1-figure supplement 1-source data 1.jpg]

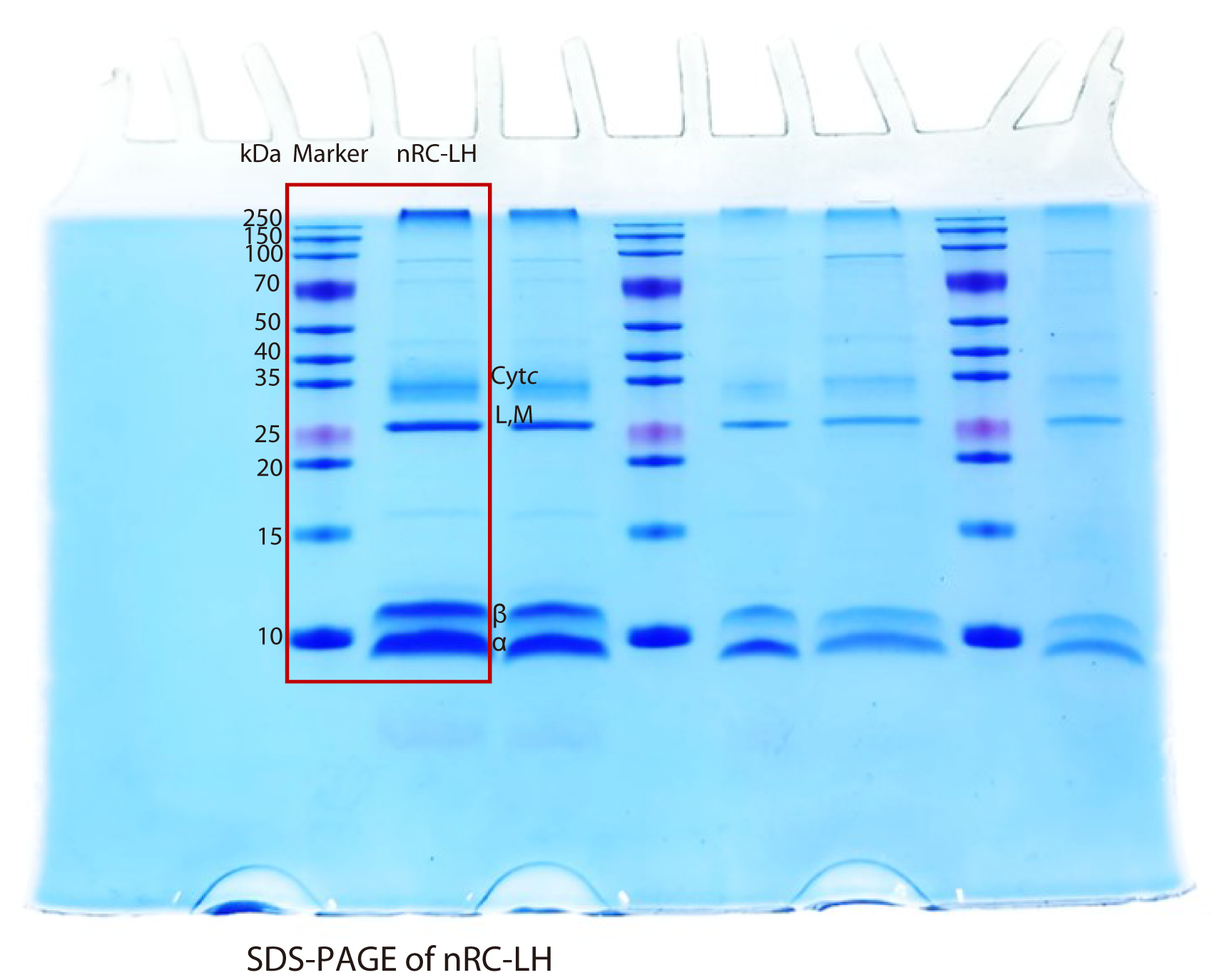

Supplement: Figure 1—figure supplement 1—source data 2. [file elife-88951-fig1-figsupp1-data2.zip › Figure 1-figure supplement 1-source data 2 with label.tif]

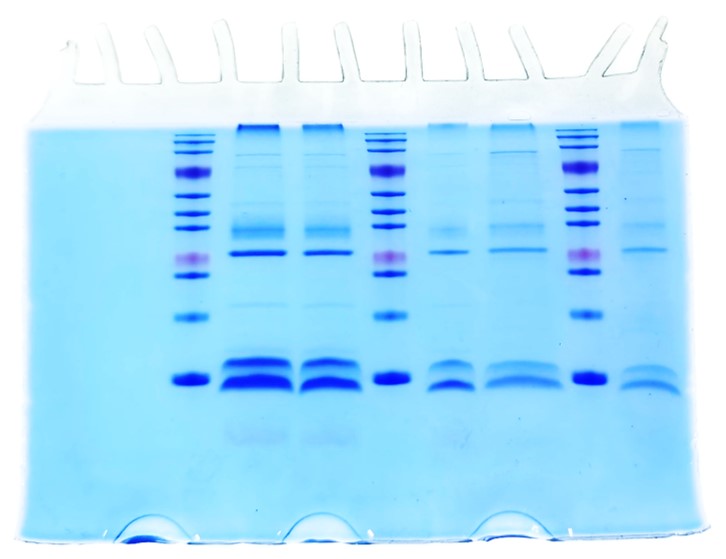

Supplement: Figure 1—figure supplement 1—source data 2. [file elife-88951-fig1-figsupp1-data2.zip › Figure 1-figure supplement 1-source data 2.jpg]

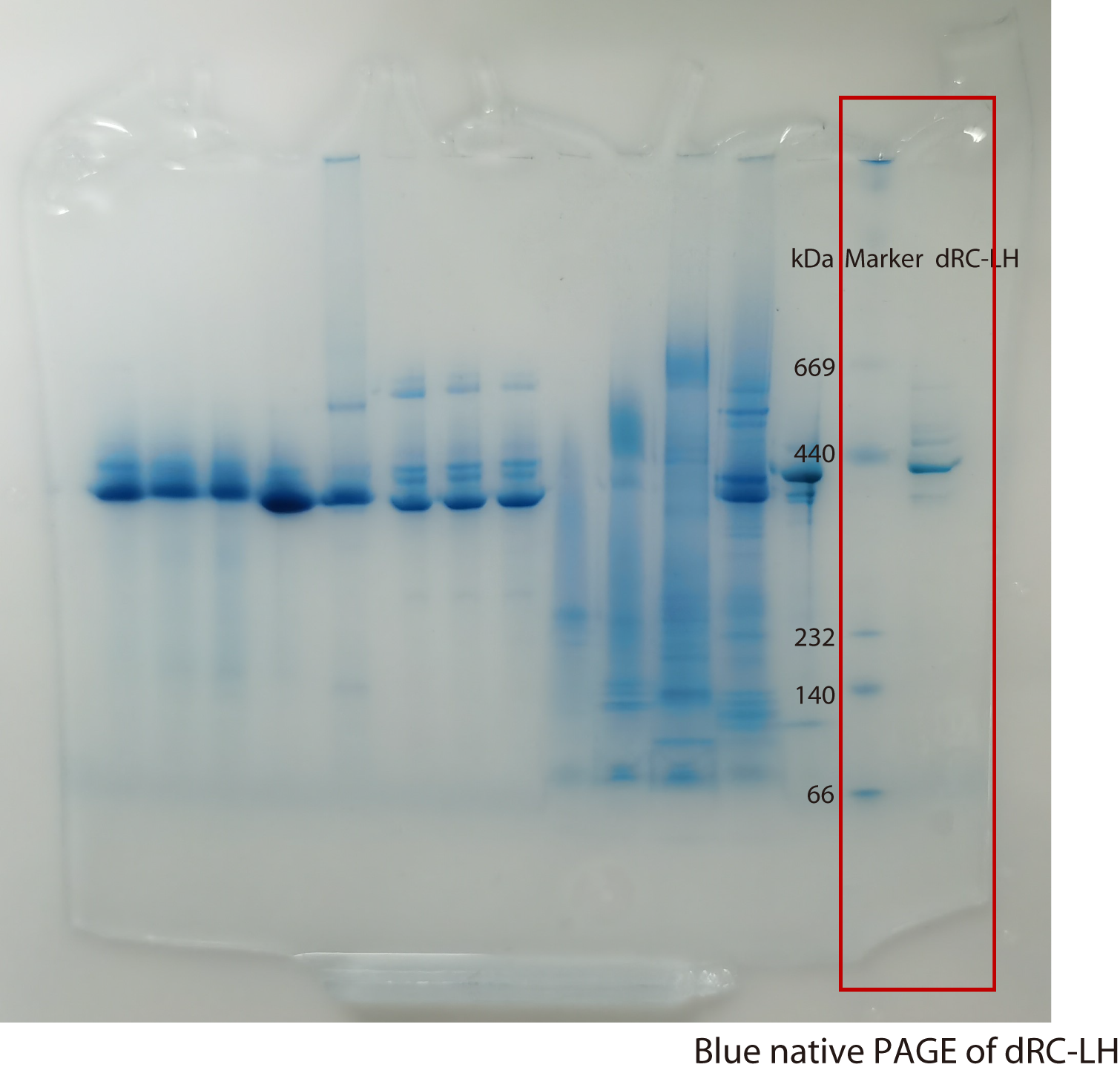

Supplement: Figure 1—figure supplement 1—source data 3. [file elife-88951-fig1-figsupp1-data3.zip › Figure 1-figure supplement 1-source data 3 with label.tif]

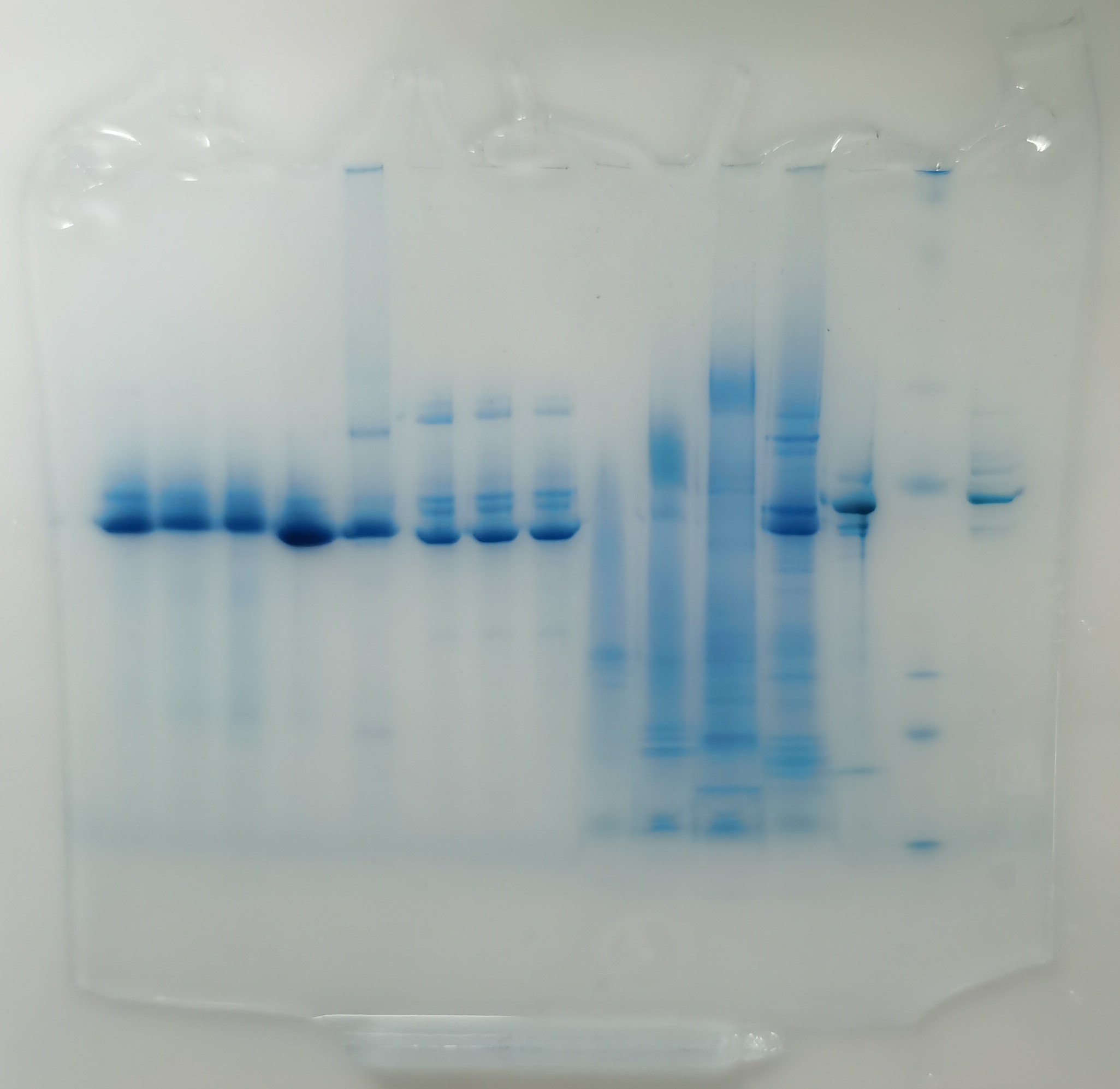

Supplement: Figure 1—figure supplement 1—source data 3. [file elife-88951-fig1-figsupp1-data3.zip › Figure 1-figure supplement 1-source data 3.jpg]

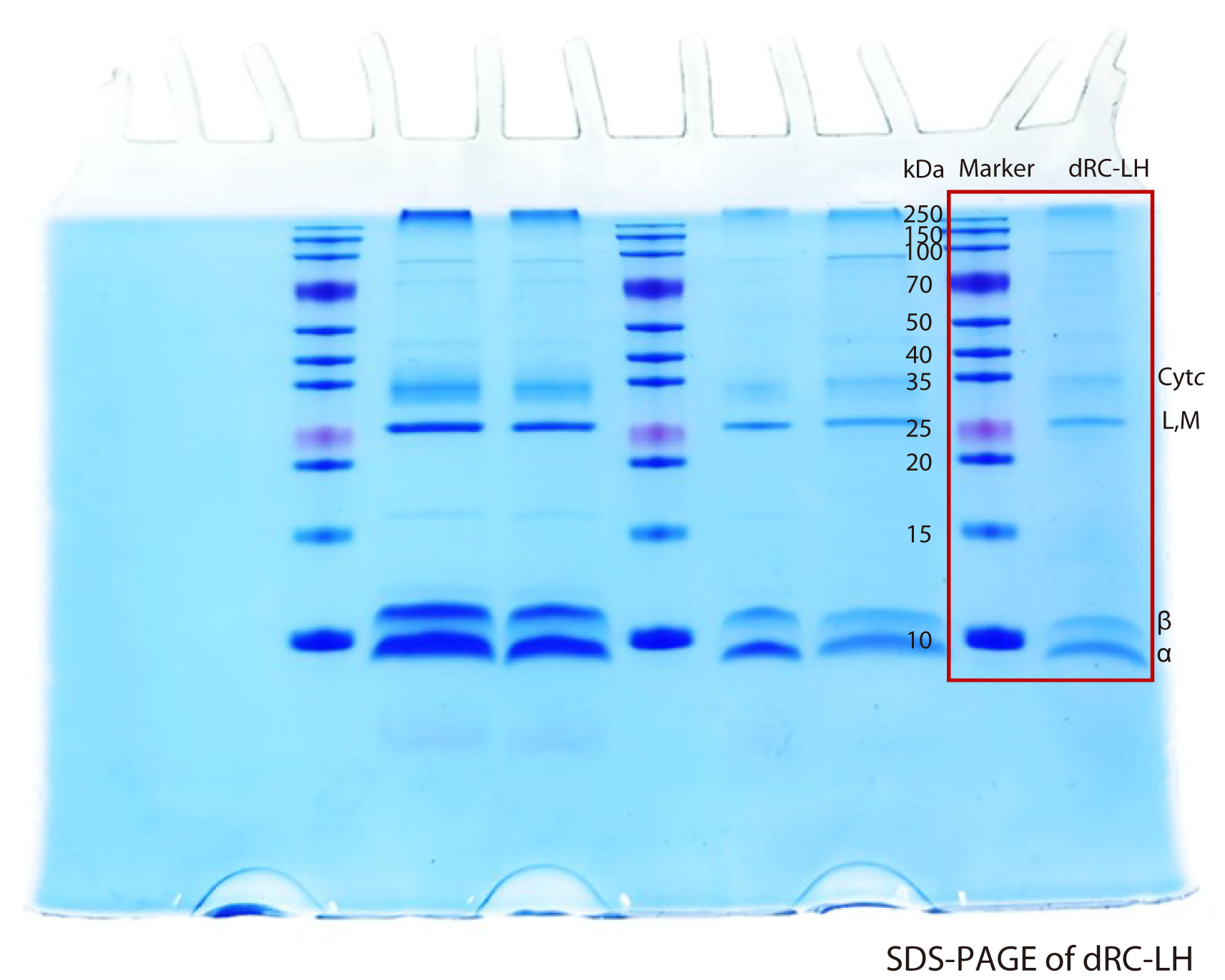

Supplement: Figure 1—figure supplement 1—source data 4. [file elife-88951-fig1-figsupp1-data4.zip › Figure 1-figure supplement 1-source data 4 with label.tif]
